# New Appliances and Things Medical

**Published:** 1906-09-08

**Authors:** 


### NEW APPLIANCES AND THINGS MEDICAL.

**[We shall be glad to receive at our Office, 28 & 29 Southampton Street, Strand, London, W.C., from the manufacturers, specimens of all new preparations and appliances which may be brought out from time to time.]**

### FRAME FOOD COCOA.

(Frame Food Co., Ltd., Standen Road, Southfields, London, S.W.)

As we understand it, this preparation consists of frame food to which cocoa in certain proportions has been added. The peculiarity of frame food, whch is, of course, a cereal food, is that it consists of the so-called frame food extract which is prepared not only from the body of the grain, but also from the bran. The bran is boiled with water under high pressure, the result of which is to break down most of its cellulose and to extract from it a large quantity of its mineral and nitrogenous constituents. A portion also of the carbo-hydrate is rendered soluble. Frame food diet consists essentially of wheaten flour to which some of this bran extract is added. The bran extract fortifies the food especially in nitrogenous constituents, although the exact nature of these nitrogenous substances is not known. The mineral constituents are also somewhat increased, especially the phosphates. The preparation also contains some unaltered starch, and this must be remembered. Frame food cocoa is prepared by adding one teaspoonful of the powder to a breakfast cup of water or milk and water, and adding sugar to taste; by boiling for a few minutes the preparation thickens slightly. The beverage thus prepared is pleasant, nutritious, and invigorating, and we think distinctly to be recommended.

### MARMITE.

(The Marmite Food Extract Company, Limited, 40 Mincing Lane, London, E.C.).

We have received two pots of marmite, one flavoured and one unflavoured. Marmite is essentially an extract obtained from yeast. The chemical composition of yeast is exceedingly attractive, from the point of view of nutrition, to the physiological chemist, and hence it is not to be wondered at that attempts have been made to utilise the nutritive constituents of spent yeast. If we were to judge from chemical data alone yeast would be one of the most nutritious substances in existence, being especially rich in the most expensive element of our diet—namely, nitrogen. Unfortunately, however, all is not gold that glitters, and much of the nitrogenous substance in yeast, although chemically *sans reproche*, is physiologically exceedingly indigestible. In addition, however, to its nitrogenous constituents yeast contains fats, phosphorated fats, and carbo-hydrates including glycogen. When we have made all deductions for the indigestible parts of yeast there remains obtainable from it both a stimulating and nutritious extract, and this has been put upon the English market under the name of "Marmite." We are already aware of two extracts other than marmite prepared from yeast—namely, sitogen and ovos. Marmite contains approximately 6 per cent. of nitrogen and about 9 per cent. of albumoses. The remaining nitrogen is present in the form of nucleins and amido-bodies, substances having physiological effects allied to those produced by the extractives of meat. The price of marmite owing to the cheapness of its source is very low; in fact, comes out at approximately 3d. per oz. It is exceedingly useful for strengthening and flavouring soups and gravies, and could, we think, with advantage find an extended use in invalid dietetics; this is more especially the case as marmite is now put up in a most convenient form for sick-room use or for travelling—namely, in bouillon tubes. Half one of these tubes, which are supplied in tins, makes an excellent cup of bouillon.

### COSENZA'S LEMON ZEST.

(Cosenza and Co., 95 Wigmore Street, London, W.)

We have received a specimen of this excellent and ingenious preparation. It is sent out in a peculiar form of twin bottle possessing two tube stoppers similar to those in use for the essences of this well-known firm. The directions for use are that a few drops from each stopper should be poured into a glass which must be subsequently filled up with water or soda-water and sweetened to taste. "The Zest" possesses a sharp and characteristic taste of fresh lemons, and when used as above makes a most refreshing summer beverage. It can also be used for cooking purposes as a lemon essence and is, in our experience, eminently satisfactory. We think the preparation to be highly recommended.

